# Mapping audiovisual content providers and resources in Greece

**DOI:** 10.1007/s00799-022-00321-6

**Published:** 2022-02-03

**Authors:** Afrodite Malliari, Ilias Nitsos, Sofia Zapounidou, Stavros Doropoulos

**Affiliations:** 1 International Hellenic University, Sindos, Greece; 2 Aristotle University of Thessaloniki, Thessaloníki, 54124 Greece; 3 Datascouting, Vakchou 30, Thessaloníki, Greece

**Keywords:** Audiovisual providers, Audiovisual resources, Greece, Open access

## Abstract

In Greece, there are many audiovisual resources available on the Internet that interest scientists and the general public. Although freely available, finding such resources often becomes a challenging task, because they are hosted on scattered websites and in different types/formats. These websites usually offer limited search options; at the same time, there is no aggregation service for audiovisual resources, nor a national registry for such content. To meet this need, the Open AudioVisual Archives project was launched and the first step in its development is to create a dataset with open access audiovisual material. The current research creates such a dataset by applying specific selection criteria in terms of copyright and content, form/use and process/technical characteristics. The results reported in this paper show that libraries, archives, museums, universities, mass media organizations, governmental and non-governmental organizations are the main types of providers, but the vast majority of resources are open courses offered by universities under the “Creative Commons” license. Providers have significant differences in terms of their collection management capabilities. Most of them do not own any kind of publishing infrastructure and use commercial streaming services, such as YouTube. In terms of metadata policy, most of the providers use application profiles instead of international metadata schemas.

## Introduction

Nowadays, there is an abundance of websites and digital libraries with informative content (not literary or artistic) that interests scientists and the general public. Despite the online presence, finding such resources often becomes a challenging task due to the existence of scattered websites hosting different types of resources, in different languages, and described with different metadata. These challenges escalate when audiovisual resources are involved. The management and upload of audiovisual resources demands higher costs and copyright issues “limiting [their] broader dissemination” [4].

Aggregation services sought to solve the problem of resource fragmentation by providing researchers with a single point of access, and by developing common standards and practices shared among their data partners. Additionally, national registry services have tried to record the institutions that create and/or hold audiovisual collections. In Greece, there is no aggregation service for audiovisual resources, nor a national registry. On the other hand, there are many audiovisual resources available online that are not searchable and easily findable. To meet this need, the Open AudioVisual Archives (OAVA) project has been launched. It is an EU co-financed project that aims to develop the OAVA platform, an aggregation service for audiovisual resources in Greece. The OAVA platform will provide access to the aggregate metadata of audiovisual material as well as to its searchable content. This will be achieved through the application of deep learning models and the development of algorithms that perform Automatic Speech Recognition in Greek and in English.

Toward the development of the OAVA platform, the first step is to create a dataset with open access audiovisual material that meets specific criteria. To identify standards and existing criteria sets, this paper first gives an overview of well-known aggregation services and national registries for audiovisual resources. Based on the findings, the paper determines the methodology and the criteria for the creation of the OAVA dataset. Search and selection processes took place between October 2020 and March 2021. The paper presents the results of this effort with useful findings about audiovisual resource management in Greece.

## Literature review

As already mentioned, the first step toward the development of the OAVA platform is to define the criteria that will be used to create the dataset. Thus, aggregation services and national registries are reviewed. Related initiatives in Greece are also presented. We conclude the literature review with a short review of selection criteria for the development of online collections.

### Aggregation services and national registries

Europeana is supported by the European Commission aiming at Europe’s cultural heritage hosted in the collections of European galleries, libraries, archives, and museums (GLAMs) [9]. Europeana has developed the Europeana Publishing Framework [8] to help European cultural heritage institutions share their collections. Image and text resources make up 97% of Europeana’s total content, while audiovisual resources make up the remaining 3% [4]. Europeana partners include the European Film Gateway,^1^ the EUScreen^2^ project and its successor, EUScreenXL. One thing to note in the case of audiovisual resources is that the criteria for data partners change slightly due to the characteristics of the format. Having a digital file format available on the Internet via a permanent URL is considered a prerequisite. [31]. Regarding metadata, legacy and local metadata schemas were converted to the Europeana Data Model using the MINT^3^ tool; content selection criteria were not restrictive [38]. Examples of resource types are TV programs and series, movies, news, entertaining programs, movies or series of documentary character, advertisements, radio programs, homemade videos, etc. Copyright is a challenging issue especially for audiovisual resources. Thus, the EU carried out another project related to orphan audiovisual works. The FORWARD project was initiated in 2013 and lasted until 2017 with the aim to “create a permanent registry for AV Orphan Works” [33].

The audiovisual heritage of the UK has been documented in various books and projects. A well-known monograph is “The Researcher’s Guide: Film, Television, Radio, and Related Documentation Collections in the UK” [2]. “Film Archives UK” is a limited company in UK that focuses on the development of the country’s public-sector film archives. To achieve its goals, the company collaborates with regional film archives, such as the National Library of Scotland’s Moving Image Archive and the National Screen & Sound Archive of Wales [16]. In Switzerland, Memobase is an association for the preservation of the audiovisual heritage of Switzerland, supported by the Swiss Confederation. It aggregates metadata for audiovisual resources, i.e., photographs, films, sound, and video, from 60 Swiss memory institutions [17]. A recent assessment of the progress of digitization, online accessibility, and digital preservation of cultural material in the European Union has shown that digitization of audiovisual heritage has a high priority in 13 EU countries, namely Belgium, Czechia, Germany, Greece, Italy, Latvia, Malta, Austria, Poland, Hungary, Slovakia, Finland, and Sweden [4]. According to [5], the COVID-19 pandemic revealed the need for more audiovisual resources that can be used in collaborative environments and online activities.

Trove is a collaboration between the National Library of Australia and several Australian organizations. It aggregates content from GLAM institutions in Australia, mainly “libraries, museums, galleries, the media, government and community organisations” [37]. They must: (a) provide the XML sitemap for their websites, (b) use aggregation technologies, such as the OAI-PMH protocol and APIs, and (c) describe the provided resources using either the Trove Data Dictionary or simple metadata schemas, such as the Dublin Core [36]. The audiovisual content aggregated by Trove is categorized as “Music, Audio & Video” content. At the end of May 2021, this category included 2.303.226 audiovisual resources (video, sound, and audio books),^4^ only a small percentage of the 6 billion digital items available through Trove [37]. Australia’s “National Registry of Audiovisual Collections” [25] includes collections from a variety of providers, including “libraries and museums, community groups, political parties, historical societies, research centers, film societies, broadcasters, distributors, production companies and foreign legations, as well as individual collectors” [24].

The “Digital New Zealand” (DigitalNZ) service was launched by the National Library of New Zealand in 2008 with the aim of aggregating all digital material related to the country, and providing a one-stop point of access. It currently has more than 30 million resources, of which only 478.500 are audio and video resources. The DigitalNZ harvests metadata from content partners, structures the metadata, and then makes it available through its API [26]. In the event that a content partner simply uploads their digital collection to a web page, the DigitalNZ service may harvest metadata and resources by scraping the metadata in the HTML web pages [28]. The DigitalNZ service helps aspiring partners in their decisions for digitizing their material (Make it Digital program) and even provides a shared repository for partners that have small collections and no repository infrastructure [27].

The Digital Public Library of America—DPLA, is a nonprofit organization that utilized the know-how of both Europeana and Trove Australia. As far as selection criteria are concerned, it focuses on exploiting intermediate providers—Hubs, which will gather and prepare the material from cooperating institutions. Hubs must initially submit 50,000 unique records. They are also responsible for ensuring that good operation and viability prerequisites are met [6]. Criteria related to audiovisual content include the level of description, the existence of metadata, ownership, and usage rights [7]. The DPLA does not exclude audiovisual material hosted in commercial streaming services, such as YouTube and Vimeo, as long as there is a copyright statement, and no advertisements are displayed. It is worth noting that the DLPA does not collect audio transcriptions into text.

The National Digital Library of India (NDLI) is a relatively new aggregation service “with a vision to turn NDLI into a National Knowledge Asset” [21]. The NDLI aggregates metadata from a variety of sources and normalizes them for integration using the NDLI metadata schema [29]. With regard to rights, the NDLI promotes the use of Rights Statements among its content partners [20]. The NDLI currently aggregates more than 68 million of textual documents and 1 million of images. Video and audio resources are nearly 866.000.^5^

In Greece, the National Documentation Centre acts as the Greek National Aggregator for Europeana. It has recently published a set of best practice guidelines for Greek GLAM institutions wishing to share their collections on Europeana via the national aggregation service called SearchCulture^6^ [23]. These guidelines focus on the Europeana Data Model, licensing issues, and technical characteristics of the digital files. In June 2021, the SearchCulture aggregation service provided access to nearly 2.500 audiovisual resources from galleries, archives, libraries, museums, and research institutions. This is less than 1% compared to the 717.700 resources offered by the service in total.

In 2006, the Hellenic National Audiovisual Archive (HNAA) was founded collecting audiovisual resources from different providers, mostly media organizations. One of its tasks was to create a registry of audiovisual archives and collections in Greece. A questionnaire was sent to universities, research institutes, libraries, archives, museums, cultural festivals, public organizations, and collectors. Preliminary results were presented in 2010 [13]; unfortunately, this project was never completed because the HNAA stopped operating in 2011. The National Centre of Audiovisual Media and Communication EKOME, established in 2015, aims to protect, support, and promote public and private initiatives “in the field of audiovisual media and communication in Greece” [19]. Based on the finding that audiovisual resources are produced and stored in different electronic environments, the EKOME is organizing the creation of a National Registry of Audiovisual Archives that aims to register all institutions holding audiovisual archives. The National Registry has not been created yet. Currently, the EKOME focuses on “attracting investment through the production of films, television series, documentaries, animation, and digital games in Greece” [19].

### Selection criteria for developing collections

All these registries and aggregation services provide specific technical standards and licensing policies in order to collect audiovisual resources from different types of cultural, media, and research institutions [7, 10, 31, 34, 36, 38] Regarding the content of the audiovisual material, selection criteria do not apply. Providers simply decide which content may be considered eligible for inclusion. Selection criteria mostly apply in internal digitization projects or other collection development procedures. Studying the relevant literature shows that there is a high degree of homogeneity in the selection criteria described in the various studies and research papers [15]. The Digital Library Federation—Council on Library and Information Resources, published a report in 2001 with selection criteria to help libraries create “high-quality collections of free Web resources” [32]. The criteria in this report were organized in 4 categories: context, content, form/use, and process or technical. The International Federation of Library Associations and Institutions (IFLA) published in 2012 guidelines and criteria for the selection of electronic resources [12]. Criteria on content (reliability, subject, complementarity with other sources) and technical criteria were introduced, in addition to the evaluation criteria for commercial providers. NISO published a framework for the development of digital collections providing best practices on collection development, item selections, and metadata [30]. Item selection mostly focused on their technical characteristics. The American Library Association [1] published a set of criteria depending on the type of library (Public, School, Academic). The criteria were organized as general and special ones focusing mostly on content characteristics (reliability, objectivity, subject, relevance to the rest of the collection).

At the European level, the Minerva project provided useful guidelines for digitization projects [18]. The guidelines acknowledged that selection criteria differ depending on each project’s goals. Nevertheless, there are general criteria that should be considered, such as copyright and licensing issues, and accessibility. In Greece, a report with best practices for digitization projects was published by a research team at the University of Patras [11]. The selection criteria of this report included copyright, cost, and digitization process issues, retention, criteria for the organization and adequate documentation of the material to be digitized, criteria related to the entity ’s potential, the purpose of the digitization project, and the intended uses for the digitized material.

To conclude this section, well-known aggregation services of cultural content are Europeana^7^ in Europe, Digital Public Library^8^ in North America, National Digital Library of India, Trove^9^ in Australia, and DigitalNZ in New Zealand. All these five aggregation services are generic ones and present similarities to one another. They all cooperate with other institutions to aggregate content and provide policies, copyright best practices, preservation and conservation ideas regarding the aggregated content. The institutions providing their content are most often, galleries, archives, libraries, museums, as well as film archives and media institutions. Despite the generic character of the aggregation services, they all aim to aggregate audiovisual heritage resources among other types of resources. Currently, the audiovisual resources aggregated by each service are only a small fragment of the total content. The need of gathering and providing access to audiovisual material in a unified way is self-evident. National libraries play a key role in preserving a nation’s heritage. With the advent of the Internet, new types of online content are easily created, spread, and lost once a web page is removed. Thus, many national libraries have begun to play an active role in the development of national digital archives and aggregation services for the collection and conservation of online and audiovisual resources. In many cases, existing library tools, criteria, and methodologies, that have already been applied to the development of online collections, are being extended and adapted to meet the needs of the new types of content. Focusing on Greece, this need to aggregate audiovisual material is imperative due to the absence of a national searchable registry.

### Licenses for reuse

As already mentioned, the goal of the present study is to create a collection of resources that meet specific criteria and can be used in the context of open access services. Content licensing must be therefore taken into consideration, i.e., the terms and conditions of each publisher/owner/provider. In the next paragraphs, well-known copyright licenses that have been used over the Internet to enable the fair and ethical use of resources are presented. These licenses are: (a) the case of the public domain (b) Greek law (4727/2020), (c) Creative Commons Licenses, (d) Rights Statements Licenses, (e) Fair use, and (f) Terms of use of channels in audiovisual material hosting services.

Resources in the Public Domain can be used in the context of open access services. According to the Greek and European legislation, intellectual property falls into the public domain 70 years after the death of the creator of the intellectual work. This means that they can be modified and used commercially by attributing to the author, without copyright or other charges.

According to the Greek law and European Union directives, any information generated by public sector organizations is considered public information, with a few exceptions, for example, national security organizations, organizations collecting personal or other sensitive data, etc. Public information may be used for any purpose by any individual or legal entity, even for commercial purposes, without the need to obtain consent or even inform the content creator. Resources that have been created by public organizations in Greece and fall under the Greek law regarding open data and the free provision of access to public information with the possibility of reuse are also considered eligible for open access use.

All material publicly available on the Internet and licensed under any Creative Commons license is considered eligible for selection. Creative Commons licenses have been in effect since 2002 to facilitate the process of publishing content on the Internet. Content creators decide how their content may be used or reused, removing all, or most of the copyright restrictions set by the law.

Material publicly available on the Internet and licensed under a Rights Statements license is usually considered eligible for open access use. However, on a few occasions, certain licenses may pose restrictions. Rights Statements licenses are typically used by cultural organizations, which have collections of content by various authors. These licenses make it easier for collection owners to explicitly state how the content may or may not be used.

When considering the “Fair Use” of content, one must keep in mind that “Fair use” is not a license. The term “Fair Use” describes the right to use proprietary material after taking into account several factors such as:

- the service makes commercial use of the content.
- the content presents facts and informs the public.
- only part of the content is used.
- the service is obstructing commercial exploitation by the content owners.

Whether a particular use of a resource falls under the terms of “Fair Use” always remains a decision of US courts.

The case of licenses offered by hosting services is the last case discussed in this section. To publish content to a hosted service, the content creator must first accept the terms and conditions under which the content will be published. This usually means that the content creator is no longer the copyright holder or the sole copyright holder of the content, allowing for several types of reuse permitted by the hosting service. Resources hosted on such services are therefore candidates for open access platforms.

## Research

Taking into consideration that the online audiovisual material usually varies from videos and narrations regarding historical and everyday life events (e.g., wars, immigration/migration) to numerous scientific, academic, and cultural events, this research aims to create a multidisciplinary dataset with open access audiovisual resources that meet specific criteria related to copyright and content, form/use, and process/technical characteristics. This dataset will be used for the OAVA platform that is under development. The platform will provide access to the aggregated metadata of audiovisual resources and also to their searchable content. As mentioned earlier, this will be achieved through the application of deep learning models and the development of algorithms that perform Automatic Speech Recognition in Greek and in English.

### Methodology

This section focuses on the methodology used to find, evaluate, and select audiovisual resources. The goal is to create a collection of resources that can be used in the context of open access services, after taking into account the terms of use and conditions of each owner or publisher. Resources should contain informative content (not literary or artistic). Consequently, musical and artistic performances, theatrical productions, films, musical works, literary readings, videos without speech, and, finally, static images were discarded during the selection process. Due to the European and Greek National Law on Intellectual Property and Neighboring Rights, the recordings of the performances had to be excluded. In addition, recordings of music without speech were outside the scope of this project, as this project aims to convert the speech contained in the audiovisual material into searchable text.

#### Finding resources

Three methods were used to find resources: a) searching with keywords on the web, b) browsing the websites of organizations that are expected to provide audiovisual content, and c) conducting an online survey.

*Keyword searching* To create a multidisciplinary result, general keyword searches from different subject areas were applied, for example, “Local History, Documentary, Oral History, Oral Interviews, Video Collections, audio collections, Video,” etc.

*Browsing the websites of institutions* A website list was used containing entries from a wide variety of memory organizations, including archival organizations, museums, libraries, galleries, businesses, private institutions, etc. The findings of the survey of audiovisual collections in Europe [14] were taken into account to create the final candidate list. The same study showed that, in Europe, there are many small multidisciplinary organizations with important (small or medium sized) audiovisual collections, and that publishers usually maintain incomplete data and documentation for their content.

*Conducting an online survey* An online questionnaire was sent to Greek reference librarians. The aim was to determine the frequency of use of audiovisual resources and at the same time to identify these resources.

#### Selection process

After discovering candidate resources, the selection process was completed in two stages. The first stage used initial evaluation criteria (in accordance with the current literature review).

**Fig. 1:**
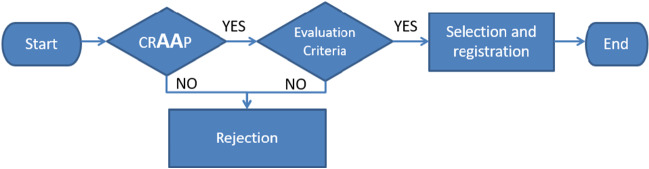
Workflow diagram

The criteria used to initially evaluate a source were: Currency, Relevance, Authority, Accuracy, and Purpose, also known as crAAp[3]. During this stage, emphasis is placed on: a) the profile of the organization that maintains the source with the audiovisual material and b) the accuracy of the content of the audiovisual material. Depending on the outcome of the initial evaluation, a website was eligible for further evaluation or was rejected, as shown in the workflow diagram (Fig. 1).

A list of initial selection criteria was compiled (Table 1) based on (a) the Digital Library Federation and Council on Library and Information Resources study [32] and (b) criteria found in other studies [7, 10, 31, 34, 36, 38]. It is worth noting that for reasons of uniformity and ease of reading, a Likert scale was chosen to describe the degree of satisfaction of each criterion: 0—Very poor, 1—Poor, 2—Barely Acceptable/Fair, 3—Good, 4—Very good.

*Context criteria* The present paper aims at compiling a collection of high-quality audiovisual resources from Greece that can be used in the context of open access services. Official organizations from the public or the private sector usually offer licenses that allow for third-party reuse (Criterion 1.1, Table 1). Multidisciplinary and (if possible) complementary resources should be used (Criterion 1.2, Table 1).

*Content criteria* The selection should be strict in terms of the validity of the content, its accuracy, authority, and uniqueness (Criteria 2.1–2.4, Table 1). In terms of completeness, coverage, and currency, a lower selection standard was applied (Criteria 2.5–2.7, Table 1). One final limitation is that the content should address the following 3 different target groups: individuals, research teams, content owners (Criterion 2.8, Table 1).

*Form/use criteria* The most important criteria in this case are criteria 3.5–3.6 (Table 1), i.e., those that determine how the content can be used by third parties and under what license the content is available. For certain providers, not all resources were selected for the dataset to comply with the terms of “Fair Use.” It is important that users do not have to go through a registration process. Criteria 3.1–3.3 that evaluate composition and site organization, navigational features, recognized standards, and appropriate technologies are also important. Criterion 3.4 for user support is rated 1 when the organization provides contact information, but no other contact option is provided. On the other hand, user support is rated 4 when chat communication is offered.

*Process or technical criteria* The set of criteria 4.1–4.3 in Table 1 can be used to assess the overall integrity of the publishing software and the infrastructure used to deliver the content. Regarding the minimum technical characteristics required for the resources, a combination has been used that takes into account: a) recommendations by the Greek National Documentation Center, b) Europeana recommendations [34], and c) the following specifications: lossless encoding and high frequency. A minimum of 16 KHz sampling rate is required, and recommended is 22KHz or ideally 44+ KHz. Bit depth must be 16 bit in case of wav files. For compressed audio in .mp3 format, 128 kbps bitrate for stereo or 64 kbps for mono is needed. The sound must be clear without ambient noise or music.

*Metadata* Providing metadata is also important and rated using the criteria 5.1–5.4 in Table 1.

After applying the selection criteria described above to a sample of resources, the rejection ratio appeared to be high. A revision was therefore necessary, and the resulting criteria used less stringent limits for inclusion in the final dataset. The specific conditions and circumstances that led to the modification of the inclusion thresholds are presented below.

**Table 1:** Selection criteria

N.	Criterion	Initial threshold	Revised threshold
1	Context		
1.1	Provenance	Resources from official organizations, public or private	Resources from official organizations, public or private
1.2	Relationship to Other Resources	Multidisciplinary and (if possible) complementary resources	Multidisciplinary and (if possible) complementary resources
2	Content	25/28	22/28
2.1	Validity	4	4
2.2	Accuracy	4	4
2.3	Authority	4	4
2.4	Uniqueness	4	4
2.5	Completeness	3	2
2.6	Coverage	3	2
2.7	Currency	3	2
2.8	Audience	Individuals, Research Teams, Content Owners	Individuals, Research Teams, Content Owners
3	Form/use	13/16	7/16
3.1	Composition and Site Organization	4	2
3.2	Navigational Features	4	2
3.3	Recognized Standards and Appropriate Technologies	4	2
3.4	User Support	1	1
3.5	Terms and Conditions	No registration required	No registration required
3.6	Rights Legitimacy	Resources with a clear description of the terms of use. Resources that license their content under Creative Commons licenses are preferred.	Resources with a clear description of the terms of use. Resources that license their content under Creative Commons licenses are preferred.
4	Process or Technical Criteria	16/16	11/16
4.1	Information Integrity	4	2
4.2	Site Integrity	4	3
4.3	System Integrity	4	3
4.4	Technical Features of the Resources	4	3
5	Metadata	14/16	5/16
5.1	Basic metadata should be included (Creator, Title, Production Year)	4	4
5.2	Metadata suitable for machine harvesting	3	0
5.3	Additional desired metadata (contributors, geographic coverage, subjects or keywords, thumbnail image)	3	0
5.4	Permalink to the location of the resource and metadata	4	1

In the case of Greek audiovisual content, it shows that many of the resources and the related websites were developed through previously funded programs that did not receive further funding for future maintenance. This affects criteria 2.6, 2.7, 4.1, and 3.1–3.4 in Table 1, respectively.

Additionally, some providers incorporate information and audiovisual resources of their collections into simple web pages and do not use specialized software such as content repositories. Simple web pages do not necessarily follow standardization and are not considered suitable for adequate audiovisual support, thus affecting criteria 4.1–4.4. In most cases, the only metadata element available for audiovisual material is the title. Criteria 5.1–5.3 were subsequently revised. Although a permalink is considered important for content aggregators such as Europeana and DPLA, it shows that many of the content providers discovered in the current study do not provide a permalink to their audiovisual material, thus affecting the criterion 5.4. The initial and revised thresholds for the selection criteria are presented in Table 1.

## Results

A dataset was created including the providers and their resources that were reviewed and evaluated with the above-mentioned criteria (Table 1). The dataset contains: provider’s name and type, channel publishing AV, resource genre, resource URL, and also the criteria (context, content, form/use, rights, process/technical criteria, metadata).The dataset can be found at Zenodo^10^ Descriptive statistics were used to summarize the characteristics of the dataset. The results of the providers’ and resources’ analysis are presented below.

### Providers

**Fig. 2:**
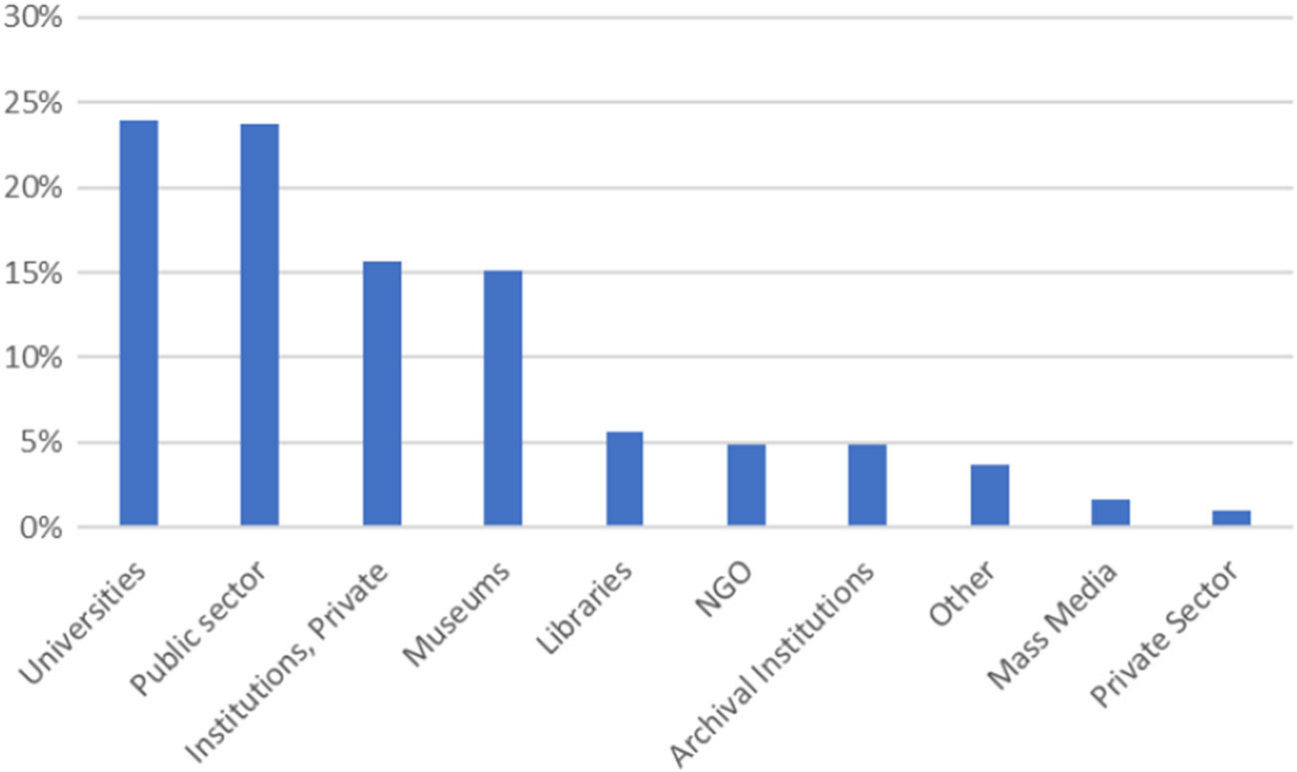
Providers’ types

Initially, 500 providers were reviewed using the crAAp test on their content and 497 of them were found to be reliable. 24% of them come from the public sector, 24% from universities, 16% from private institutions, and another 15% from museums. Details are presented in Fig. 2.

The 497 reliable providers were further reviewed and evaluated by applying on their content the selection criteria presented in the methodology section. 233 of the 497 providers were rated as eligible for inclusion in the final list of trusted providers (Table 2). It should be mentioned that 30% of them belonged to the public sector, 28% to universities, and 13% to private institutions. It is worth mentioning that the providers that were excluded from the final list (264 of the 497) shared audiovisual material that was not eligible for selection either because of its content as it was non-verbal audio, or it did not give permissions to be used from other parties.

**Table 2:** Eligible vs non-eligible providers

Type	Frequency	Percent
Non-eligible	264	53%
Eligible	233	47%

Most of the providers (70%) publishing eligible resources for the OAVA platform have a YouTube channel, while 19% of them share and promote their audiovisual material via their website (Table 3).

**Table 3:** Providers’ channels for publishing AV material

Provider	Frequency	Percent
YouTube	163	70%
Website	44	19%
Repository	19	8%
Vimeo	7	3%

In terms of metadata policy, eligible providers use either application profiles developed according to their own needs and/or the capabilities of the information systems they use or international metadata schemas like Dublin Core. As depicted in Table 4, the vast majority (97%) uses application profiles and only 2,6% uses Dublin Core. There is also one provider that uses Unimarc.

**Table 4:** Use of metadata

Metadata	Frequency	Percent
Application profiles	226	97%
Dublin Core	6	2,6%
Other (Unimarc)	1	0,4%

### Resources

**Fig. 3:**
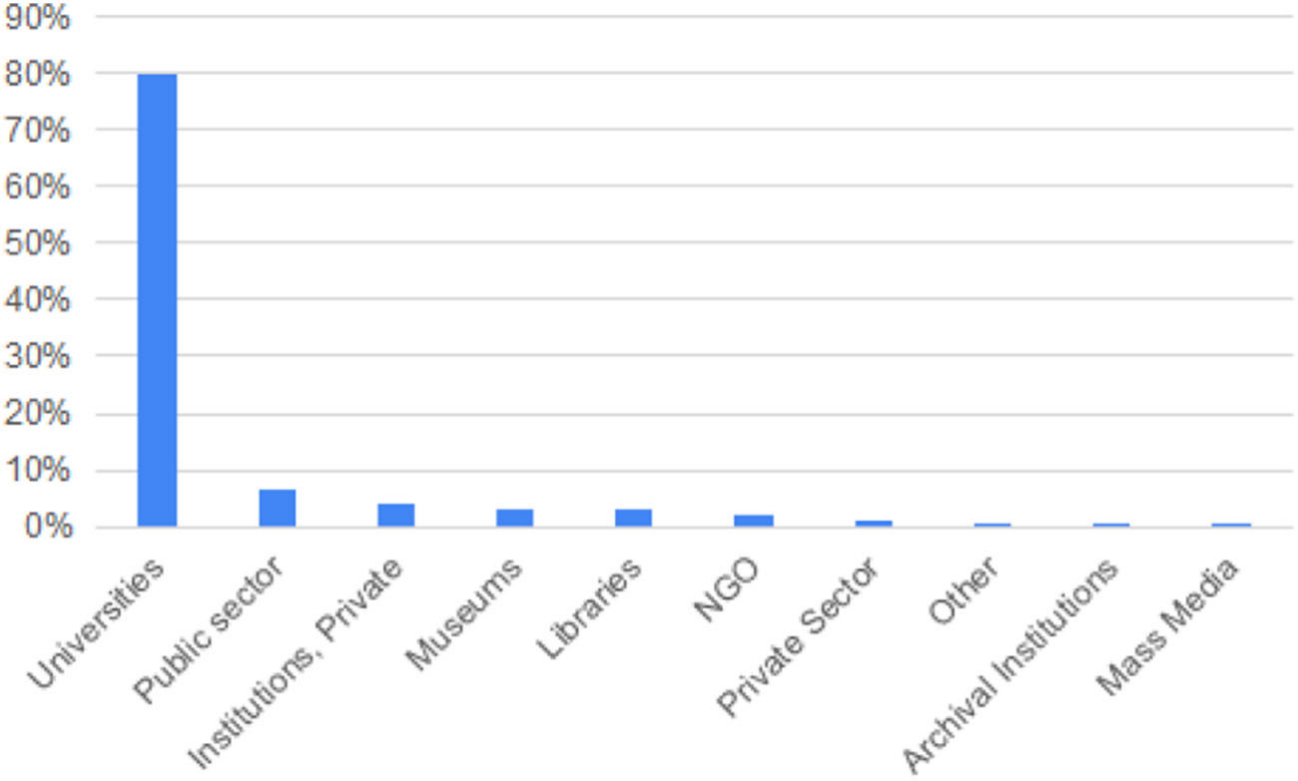
Resources per eligible providers

The 233 eligible providers offer a total of 1710 resources. 80% of these resources come from Universities, while the remaining 20% is divided to public sector, private institutions, museum, libraries, etc. (Fig. 3).

The resources have been categorized according to their genre (Table 5). There were resources related to education and training (“open courses” and “open educational material”), others related to academic and/or scientific events like webinars, lectures, etc., and others related to cultural events. Another category, “Interviews,” contains oral histories, press conferences, and similar types of recordings. “Board meetings” refer to recordings of municipality sessions. Some providers like NGOs shared audiovisual material in order to communicate and promote their missions and those resources were gathered under the genre “Campaigns.” Finally, there were audiovisual resources that were hosted in archival institutions’ repositories referring to archival material. As presented in Table 5, the vast majority of the resources (77%) are related to open courses.

**Table 5:** Resources’ genre

Genre	Freq.	Per.
Open courses	1317	77.0%
Academic/Scientific events	152	8.9%
Campaigns	93	5.4%
Cultural events	41	2.4%
Board Meetings	39	2.3%
Interviews	33	1.9%
Open educational materials	27	1.6%
Archival Material	6	0.4%
Digital Collections/Research projects	2	0.1%

The selection criteria described in the methodology section are grouped into: context, content, form/use, process or technical criteria, and metadata. In terms of “context,” each of the 233 providers was found to be in line with the objectives of the OAVA platform. Their 1710 resources gathered on average 27.20 points/grades. (The lowest was 22 and the highest 28.) Resources’ interfaces were rated on average 10.06 on a scale from 7 to 16. The process and the technical characteristics gathered on average 13.79 points. (The lowest was 11 and the highest 16.) However, in terms of metadata usage, resources received low scores (8.97 on average), with 5 being the lowest and 16 the highest.

With respect to content licensing, most of the resources (78.6%) are under “Creative Commons” license, while 20.1% under “Fair Use” license (Table 6).

**Table 6:** Resources’ licenses

License Type	Frequency	Percent
Creative Commons	1344	78.6%
Fair Use	343	20.1%
Other	21	1.2%
Rights Statement	2	0.1%

## Discussion

This study has attempted to map the audiovisual content providers in Greece and to create a collection of resources that can be used in the context of open access services. Specific criteria have been applied to reject low quality content, or content under restrictive licenses. Interesting trends have been revealed during this process. For example, significant differences have been identified between AV providers in terms of their collection management capabilities. Libraries, archives, museums, universities, governmental and non-governmental organizations, mass media organizations are among the main types of providers found in this study. Similar provider profiles are reported in other European countries as well [14].

Surprisingly, most of the providers do not own any kind of publishing infrastructure and end up using commercial streaming services, such as YouTube. The fact that many providers host their content on commercial services has a major impact on the metadata describing the resources and on licenses determining their future use. The “metadata usage” criterion in this study received the lowest score, for example, since most audiovisual resources are described by a single title. In addition, the content is licensed under the typical streaming service license, which in most cases does not comply with European and Greek copyright regulations and practices. Nevertheless, there are exceptions; universities typically own publishing infrastructure and present expertise in policy making, metadata, licensing, and preservation. Most importantly, universities have dedicated personnel and funding (usually EU funds) that ensure the sustainability of their audiovisual collections. Overall, there are a variety of institutions that produce, provide, or maintain audiovisual resources, with wide differences in staff, funds, policies, and IT infrastructure.

The maturity and expertise of universities regarding the management of collections is evident when the volume of published audiovisual resources is considered. Even though only one-third of the eligible providers are universities, their contribution is 80% of the total 1710 resources. This, of course, affects the characteristics of the selected resources. Consequently, the vast majority of the resources are open courses and academic/scientific events. Trends in licensing and metadata policies are also affected by the volume of university content. Creative Commons licenses are the mostly used licensing scheme, while dedicated application profiles are used for the description of audiovisual resources.

The aforementioned contradictions and differences inevitably hinder the implementation of national policies toward the goal of maintaining and accessing audiovisual resources in Greece. A feasible solution to this problem would be for the National Library of Greece or the National Documentation Centre, that acts as the national aggregator for Europeana, to adopt practices already implemented by the National Library of New Zealand and the National Digital Library of India. The National Library of New Zealand offers a shared repository to providers with small audiovisual collections and limited human resources and/or economic funds [27]. The National Digital Library of India promotes the use of open licenses and organizes training sessions and workshops focusing on diverse collection management issues, such as IT systems, licensing, and metadata [22]. The combination of these two strategies will likely contribute to the preservation, aggregation, better description, more clear usage rights, and easier access to audiovisual resources that will ultimately enable the provision of advanced information services.

Long-term preservation of audiovisual resources in Greece is threatened by the lack of related policies. In Greece, there is no national policy for the management and long-term preservation of audiovisual resources. The Greek National Registry of audiovisual providers has not yet been implemented, and there is no reference point for the search and access to audiovisual material. The lack of nationwide policies and registration tools is expected to have a negative impact on both long-term preservation and access to audiovisual resources. Thus, institutions in Greece that may implement nationwide policies must undertake initiatives to safeguard the country’s reservoir of audiovisual resources. Examples of such institutions could be the Ministry of Culture and Sports, the Ministry of Digital Governance, the National Library of Greece, the National Documentation Centre (as the national aggregator for Europeana), and the National Centre of Audiovisual Media and Communication EKOME.

This study has suggested possible strategies for improving the collection management capabilities of smaller providers. Most certainly, these strategies need some time to be designed and applied. However, there is the current need to search and access audiovisual content [5]. Yet, data in this study revealed a) lack of licensing policies in most providers using commercial streaming services and b) low use of well-known metadata schemas. The absence of clear licenses obstructs the reuse of resources. The absence of using well-known metadata schemas makes searching and locating information almost impossible.

A service that aggregates audiovisual resources and the accompanying metadata is evidently necessary. Content licenses must be checked in advance. The OAVA platform aims to offer a one-stop point for searching and accessing audiovisual resources in Greece. In addition to aggregating and converting different metadata into a coherent schema, OAVA will offer full-text search of audiovisual resources using Automatic Speech Recognition for resources in both Greek and in English. The OAVA platform is under development, and its beta version is to be published in late 2021.

## Footnotes

1 https://www.europeanfilmgateway.eu/.

2 https://euscreen.eu/.

3 http://mint-projects.image.ntua.gr/euscreenxl.

4 The count of resources per format has been provided to the authors by the Trove support team on June 2, 2021.

5 The count took place using the NDLI’s “Browse by type” option on May 31, 2021.

6 https://www.searchculture.gr/.

7 https://www.europeana.eu.

8 https://dp.la/.

9 https://trove.nla.gov.au/.

10 https://doi.org/10.5281/zenodo.5112283.
